# Mental health status of doctors and nurses in a Nigerian tertiary hospital: A COVID-19 experience

**DOI:** 10.4102/sajpsychiatry.v28i0.1904

**Published:** 2022-10-28

**Authors:** Olufunto A. Olude, Kofoworola Odeyemi, Oluchi J. Kanma-Okafor, Oluwaseun A. Badru, Shakira A. Bashir, John O. Olusegun, Olayinka Atilola

**Affiliations:** 1 Department of Pharmacy, Federal Neuropsychiatry Hospital, Lagos, Nigeria; 2 Department of Community Health and Primary Care, Faculty of Clinical Science, University of Lagos, Lagos, Nigeria; 3 Department of Physiotherapy, Faculty of Health Sciences, Usmanu Danfodiyo University Teaching Hospital, Sokoto, Nigeria; 4 Department of Clinical Psychology, Federal Neuropsychiatry Hospital, Lagos, Nigeria; 5 Department of Behavioural Medicine, Faculty of Clinical Science, Lagos State University College of Medicine, Lagos, Nigeria

**Keywords:** depression, anxiety, stress, healthcare professional, COVID-19

## Abstract

**Background:**

Healthcare professionals (HCPs) working to save lives during the coronavirus disease 2019 (COVID-19) pandemic are under tremendous physical and psychological pressure, therefore facing the risk of developing challenges with mental health.

**Aim:**

This study aimed primarily to determine the prevalence and factors associated with depression, anxiety and stress among HCPs in a tertiary hospital in Lagos State during the COVID-19 pandemic.

**Setting:**

Lagos State University Teaching Hospital (LASUTH), Ikeja, Lagos, Nigeria.

**Methods:**

This was a descriptive cross-sectional study conducted between June and July 2021 among 1452 doctors and nurses in LASUTH, Ikeja, Lagos, Nigeria, selected by the multistage sampling method. Depression, anxiety and stress were assessed using the Patient Health Questionnaire, Generalised Anxiety Disorder and Perceived Stress Scale, respectively.

**Results:**

The majority of respondents were female (72.5%), with two-thirds being nurses. The prevalence of depression, anxiety and stress was 9.8%, 5.0% and 62.4%, respectively. Nurses showed a higher prevalence of these mental health conditions as compared with doctors. Younger HCPs, nurses, those that lost a colleague to COVID-19, and those whose family members were infected with COVID-19 were more likely to be depressed. Nurses and those afraid of being infected were more likely to experience anxiety. Younger HCPs, nurses, history of anxiety and/or depression and previous COVID-19 infection were identified as factors associated with stress.

**Conclusion:**

Stress was the most prevalent mental health condition with nurses being the most affected of the HCPs and at a greater risk of developing challenges with mental health. Psychosocial interventions and stress management techniques are recommended to minimise the risks.

**Contribution:**

This study adds to the few studies on the mental health of HCPs during COVID-19 and calls for in-depth surveys to understand psychosocial challenges among HCPs in Nigeria.

## Introduction

The first case of coronavirus disease 2019 (COVID-19) broke out in China at the end of December 2019, leading to a pandemic.^1,2^ The pandemic posed a major challenge socially, economically and above all, psychologically worldwide, with healthcare professionals (HCPs) undoubtedly remaining one of the most vulnerable groups.^3^

While the world faced lockdowns, with reduced activity and implementation of social distancing, to reduce chances of community transmission, HCPs performed their duties at the frontline, with continuous pressure to attend to the needs of patients who were heavily dependent on them.^3,4^

The clinical HCPs, particularly doctors and nurses who worked closely to treat COVID-19, had continuous fear of infection and witnessed frequent mortality^2^ and, as such, were at greater risk of developing symptoms and challenges of a psychological nature such as anxiety, panic or other stress-related disorders.^5,6^

Research has shown that HCPs were significantly at risk of mental health problems during the pandemic. For instance, in China, half (50.0%) and 45.0% of HCPs were found to be depressed and anxious, respectively,^7^ while about 5 in 10 HCPs (48.0%) were reported to be depressed and one-third experienced anxiety, and 57.0% were stressed in the United States.^8^ Similar patterns have been reported in other studies.^9,10,11,12^ Healthcare professionals in Africa have equally shown evidence of COVID-19 related mental health issues. Nine in 10 HCPs were reported to be depressed, anxious or stressed in Egypt.^13^ In Ghana and Tunisia, 21% – 30.0% of HCPs were reported to be depressed, 24.3% – 27.8% anxious and 8.2% – 18.6% stressed.^14,15^

Healthcare professionals have adopted different coping strategies such as optimism and resilience to manage psychological stress during pandemic, and these strategies have shown to be effective.^5^ Similarly, during COVID-19 pandemic, HCPs were reported to have adopted strategies such as engaging in health promotion activities such as resting and consuming balanced diet and learning more about COVID-19 to cope with the pandemic.^16^

There is a dearth of studies on the mental health of HCPs in Nigeria, even though Nigeria ranks among the top five countries in Africa with regard to COVID-19 infections.^17^ There is a need to quantify the magnitude of the symptoms of depression, anxiety and stress among clinical HCPs, particularly the doctors and nurses in Nigeria.

Therefore, this study aimed to assess the prevalence and factors associated with depression, anxiety and stress among HCPs in a tertiary hospital in Lagos State during the COVID-19 pandemic as well as the coping strategies adopted by these HCPs.

## Research methods and design

### Study design, setting and sampling technique

A descriptive cross-sectional study design was adopted in this study. Doctors (consultants and medical residents) and nurses at the Lagos State University Teaching Hospital (LASUTH), Ikeja Lagos State, were surveyed. Lagos State University Teaching Hospital is a tertiary institution owned by the Lagos State Government with several departments such as paediatrics and surgery. Data were collected between June and July 2021 via a self-administered questionnaire. Doctors and nurses that worked in the facility as of March 2020 were eligible to participate; interns and house officers were excluded as they were not working in the study location as of the beginning of the pandemic.

### Sample size calculation and sampling technique

The sample size was calculated with the Cochrane formula (*Z*^2^[pq]/*d*^2^),^18^ where *Z* was 1.96 at a 95.0% confidence interval (CI), and ‘*p*’ was the proportion of anxiety among healthcare workers in a previous study conducted in Ghana (27.8%),^15^ *q* = 1–*p*, and ‘*d*’ was the level of precision at 0.05.

The calculated sample size was 308, with an additional 10% included to correct for non-response, yielding a sample size of 340 HCPs. According to the hospital records, a total of 1452 doctors and nurses (511 doctors, 941 nurses) worked in the 14 departments (such as psychiatry internal medicine, etc.) that make up LASUTH. By proportionate sampling, the HCPs to be selected from each department were determined. Systematic random sampling was employed from the sampling frame of HCPs. Doctors and nurses were selected for all departments. In all, 120 doctors and 220 nurses (a total of 340 HCPs) were recruited for this study.

### Data collection

Sociodemographic characteristics such as age, gender, marital status and occupation were obtained and work-related details such as years of employment and history of anxiety. The Patient Health Questionnaire 9 (PHQ-9), Generalised Anxiety Disorder 7 (GAD-7) and Perceived Stress Scale (PSS) were used to measure depression, anxiety and stress, respectively, while the coping strategy subscale of the COVID-19 questionnaire was used to assess how the HCPs coped with the infection. Other variables include access and adequacy of personal protective equipment (PPE), exposure to COVID-19, the fear of transmitting COVID-19 to colleagues and family and the history of depression, anxiety and comorbidity were sought from the respondents.

### Measures

Depression was measured with the PHQ-9, which has been previously used to assess depression among healthcare workers and the general population.^12,19,20^ The nine-item tool has a four-point Likert scale (not at all = 0 to nearly every day = 3). The scale contains questions such as, ‘feeling down, depressed, or hopeless’ and ‘poor appetite or overeating’. The score range of the PHQ-9 is 0–27, HCPs that score ≥ 10 were considered to be depressed, while those that score below 10 were considered not to be depressed.^21,22^ The PHQ-9 has a Cronbach alpha reliability of 0.84–0.89.^20,22^ In this present study, the Cronbach alpha reliability of the PHQ-9 was 0.868, which is considered very good according to,^23^ and the area under the curve (AUC) was 0.795 (95% CI: 0.713–0.877), which is considered good.^24^

Anxiety was measured with the GAD-7, which is a seven-item screening measure with a four-point Likert scale (not at all = 0 to nearly every day = 3). The scale contains questions such as ‘feeling nervous, anxious, or on edge’ and ‘feeling afraid, as if something awful might happen’. The GAD-7 score ranges between 0 and 21. Healthcare professionals that score ≥ 10 were considered to be anxious, while those that score less than 10 were considered not to be anxious.^21,22^ GAD-7 has been previously used to assess anxiety in several studies with a Cronbach alpha reliability range of 0.80–0.92.^12,22,25^ The reliability of the GAD-7 in this present study was very good^23^ at 0.876, and the AUC was fair^24^ with 0.689 (95% CI: 0.566–0.813).

The PSS was used to measure stress among the HCPs. The 10-item tool is measured on a five-point Likert scale (from never to very often) and has a score range of 0–40. Four positively worded items (4, 5, 7, 8) were reversed (0 = 4, 1 = 3, 2 = 2, 3 = 1 and 4 = 0). The scale contains questions such as ‘in the past month, how often have you felt that you were unable to control the important things in your life’ and ‘in the past month, how often have you felt nervous and stressed’. Healthcare professionals that score ≥ 14 were considered to be stressed.^12^ The Cronbach alpha reported by an earlier study was 0.82,^12^ while the Cronbach alpha was low^26^ in this present study with 0.575. The AUC for PSS was good^24^ with 0.718 (95% CI: 0.661–0.774).

The coping strategies subscale of the COVID-19 questionnaire developed by Zhang et al. was adapted to assess coping strategy with a reliability of 0.77.^16^ The reliability of the tool was 0.713 in this present study. Twelve statements were used to assess coping strategies, such as ‘I actively learn about COVID-19 (symptoms, route of transmission)?’ and ‘I keep busy to refrain from thinking about the epidemic?’. The items were placed on a five-point Likert scale (1 = Never to 5 = Always). However, our concern was the individual items as they are relevant to this present study.

### Data analysis

The SPSS version 26 was used to analyse the data. Simple frequency and means (standard deviation) were used to describe the variables. For binary analysis, Pearson’s chi-square or Fisher’s texts, where appropriate, were used. Spearman’s correlation was used to test the association between the dependent variables because of skewness. Binary logistic regression was used at multivariate analysis to control for confounders. Before then, multicollinearity between the independent variable was tested with the variance inflation factor (VIF). No evidence of collinearity was found as all the VIF were < 2.0. Variables with a *p*-value of ≤ 0.1 at bivariate analysis were considered for multivariate analysis, while regression analysis was computed at a 95% CI. However, some potential confounders were forced into a model, if necessary, for statistical relevance as suggested by Bursac et al.^27^ Hosmer-Lemeshow test was used to assess the fitness of the models. Nagelkerke *R*^2^ was deployed to measure the level of variance explained by the independent variables. Later, AUC was used to assess the predictability power of the dependent variables.

### Ethical considerations

Ethical approval was obtained from the Health Research Ethics Committee (HREC) of the Lagos University Teaching Hospital (HREC Assigned NO: ADM/DSCT/HREC/APP/4331 and LASUTH with reference number LREC/00/10/1598). Written informed consent was sought from the participants after an explicit explanation of the study purpose. Confidentiality was maintained, and participants were allowed to withdraw from the study at any time. Participation was purely voluntary; non-participation was without consequence.

## Results

### Sociodemographic, history of anxiety and/or depression and comorbidity

Of the 340 questionnaires distributed, 338 were fit for data analysis giving a response rate of 99.4%. The majority (45.9%) of the health workers were between the ages of 30 years and 39 years, with a mean ± standard deviation of 34.03 ± 8.03 years. The majority (72.5%) were females, and two-thirds (64.5%) were nurses. More than half (58.6%) were married and 41.4% were not married. The majority of the HCPs were Christians (75.7%), and one-fifth lived alone. The mean length of working in the hospital was 6.53 ± 5.96 years, and the majority (58.3%) had worked up to five years in the facility, while only 18% had worked for more than a decade. While 9 in 10 of the HCPs had access to PPE, 61.5% classified the PPE as inadequate. The average working days for the HCPs was 3.66 ± 1.32 during the peak of the pandemic, while the majority (55.9%) worked between three and four days weekly. The majority (89.1%) had no comorbidity, and more than one-fifth (23.1%) have a history of depression or anxiety (Table 1).

**TABLE 1:** Sociodemographic characteristics, history of anxiety and/or depression and comorbidity (*n* = 338).

Variables	Frequency	Percentage
**Age (years)**		
20–29	109	32.2
30–39	155	45.9
≥ 40	74	21.9
Mean Age ± SD	34.03 ± 8.03	-
**Gender**		
Male	93	27.5
Female	245	72.5
**Occupation**		
Doctor	120	35.5
Nurse	218	64.5
**Marital status**		
Married	198	58.6
Not-married	140	41.4
**Religion**		
Christianity	256	75.7
Islam and Others	82	24.3
**Whom you live with**		
Alone	67	19.8
With family	271	80.2
**Years of employment**		
1–5	197	58.3
6–10	80	23.7
>10	61	18.0
Mean years ± SD	6.53 ± 5.96	-
**Days worked per week during pandemic†**		
1–2	59	18.0
3–4	184	55.9
≥ 5	86	26.1
Mean days ± SD	3.66 ± 1.32	-
**Comorbidities**		
None	301	89.1
Cardiovascular disease	18	5.3
Respiratory illness	10	2.9
Diabetes	9	2.7
**History of anxiety and/or depression**		
Yes	78	23.1
No	260	76.9

SD, standard deviation.

† , Variable less than the total population.

Seven in 10 of the HCPs had contact with suspected cases, while more than half (53.3%) had contact with a confirmed case of COVID-19. One in six (16.9%) of the HCPs contracted COVID-19. Many (79.9%) had a colleague infected with the virus and more than one-third (35.2%) of these colleagues died from COVID-19 infection. One in five (20.4%) of the HCPs had a family member that contracted COVID-19, while 5.6% of the HCPs lost a family member to COVID-19. A large proportion (77.5%) of the HCPs are willing to work as frontline staff (Table 2).

**TABLE 2:** Personal protective equipment, contact with COVID-19 cases and infection (*n* = 338).

Variables	Frequency	Percentage
**Access to PPE**		
Yes	307	90.8
No	31	9.2
**Adequacy of PPE**		
Adequate	130	38.5
Inadequate	208	61.5
**Levels of the protective measures**		
Adequate	118	34.9
Insufficient	214	63.3
None	6	1.8
**Contact with a suspected case**		
Yes	237	70.1
No	101	29.9
**Contact with confirmed cases of COVID-19**		
Yes	180	53.3
No	158	46.7
**Contact with suspected or confirmed specimen**		
Yes	78	23.1
No	260	76.9
**Got infected with COVID-19**		
Yes	57	16.9
No	281	83.1
**A colleague got infected with COVID-19**		
Yes	270	79.9
No	68	20.1
**A colleague died from COVID-19**		
Yes	119	35.2
No	219	64.8
**A family member got infected with COVID-19**		
Yes	69	20.4
No	269	79.6
**A family member died of COVID-19**		
Yes	19	5.6
No	319	94.4
**Afraid of getting infected with COVID-19**		
Yes	299	88.5
No	39	11.5
**Willingness to work as a frontline staff**		
Yes	262	77.5
No	76	22.5

PPE, personal protective equipment

### Prevalence of depression, anxiety, stress and coping strategy and correlation analysis

The prevalence of depression, anxiety and stress was 9.8%, 5.0% and 62.4%, respectively. Several coping strategies were employed by the HCPs to cope with stress and anxiety. For instance, 45.9% and 45.9%, respectively, learnt about COVID-19 always to cope with the pandemic, while 11% stopped watching TV to avoid COVID-19 news. To cope with the pandemic, 59.2% constantly wore face masks and routinely washed their hands. Ten per cent of employees constantly communicated their worries and needs to their supervisors, while 9.5% took adjuvant medicine, and 4.4% let their feelings out through tears. Six per cent sought psychological support from colleagues, 11.5% always sought spiritual guidance regularly, and 26.6% engaged in chatting with family and friends.

We found a significant positive association between the three dependent variables: depression and anxiety (rs = 0.683, *p* = < 0.001), depression and perceived stress (rs = 0.460, *p* = < 0.001) and anxiety and perceived stress (rs = 0.521, *p* = < 0.001).

### Factors associated with depression, anxiety and stress

Independent variables with a *p*-value of ≤ 0.1 at bivariate analysis were considered for multivariate analysis. Of the seven variables associated with depression (Table 3 and Table 4), five variables were identified as factors associated with depression among the HCPs when the regression model was fitted (Table 5). Healthcare professionals that were at least 40 years were less likely to be depressed compared with those between 20 years and 29 years (odds ratio: 0.167; 95% CI: 0.043–0.649). Nurses were 1.19 more likely (95% CI: 1.194–13.103) to be depressed than doctors; while HCPs that came in contact with specimen were 2.50 times (95% CI: 1.033–6.070) more likely to be depressed. Healthcare professionals whose colleague died of COVID-19 were 2.91 times (95% CI: 1.210–7.004) more likely to be depressed. Also, those whose family members were infected with COVID-19 were 2.66 times (95% CI: 1.128–6.265) more likely to be depressed. The independent variables explained 22.3% of the variance.

**TABLE 3:** Association between sociodemographic, history of anxiety and/or depression and comorbidity and the dependent variables.

Variables	Depression					Anxiety					Stress				
	No	%	Yes	%	*p*	No	%	Yes	%	*p*	No	%	Yes	%	*p*
**Age (years)**															
20–29	92	84.4	17	15.6	0.028F*	101	92.7	8	7.3	0.402F	28	25.7	81	74.3	0.003*
30–39	142	91.6	13	8.4		148	95.5	7	4.5		62	40.0	93	60.0	
≥ 40	71	95.9	3	4.1		72	97.3	2	2.7		37	50.0	37	50.0	
**Gender**															
Male	83	89.2	10	10.8	0.706	87	93.5	6	6.5	0.461	36	38.7	57	61.3	0.791
Female	222	90.6	23	9.4		234	95.5	11	4.5		91	37.1	154	62.9	
**Occupation**															
Doctor	115	95.8	5	4.2	0.010*	118	98.3	2	1.7	0.038F*	61	50.8	59	49.2	< 0.001*
Nurse	190	87.2	28	12.8		203	93.1	15	6.9		66	30.3	152	69.7	
**Marital status**															
Married	181	91.4	17	8.6	0.386	189	95.5	9	44.5	0.628	82	41.4	116	58.6	0.083*
Not-married	124	88.6	16	11.4		132	94.3	8	5.7		45	32.1	95	67.9	
**Religion**															
Christianity	234	91.4	22	8.6	0.201	244	95.3	12	4.7	0.611	97	37.9	159	62.1	0.832
Islam and Others	71	86.6	11	13.4		77	93.9	5	6.1		30	36.6	52	63.4	
**Whom you live with**															
Alone	61	91.0	6	9.0	0.803	63	94.0	4	6.0	0.754F	18	26.9	49	73.1	0.043*
With family	244	90.0	27	10.0		258	95.2	13	4.8		109	40.2	162	59.8	
**Years of employment**															
1–5	175	88.8	22	11.2	0.159F	187	94.9	10	5.1	0.787F	69	35.0	128	65.0	0.061*
6–10	71	88.8	9	11.2		75	93.8	5	6.2		27	33.8	53	66.2	
> 10	59	96.7	2	3.3		59	96.6	2	3.3		31	50.8	30	49.2	
**Days worked per week during the pandemic**															
1–2	51	86.4	8	13.6	0.320	53	89.8	6	10.2	0.108	21	35.6	38	64.4	0.679
3–4	170	92.4	14	7.6		178	96.7	6	3.3		68	37.0	116	63.0	
≥ 5	76	88.4	10	11.6		81	94.2	5	5.8		36	41.9	50	58.1	
**Comorbidities**															
None	272	90.4	29	9.6	0.531F	286	95.0	15	5.0	0.357F	110	36	191	63.5	0.624F
Cardiovascular disease	17	94.4	1	5.6		18	100.0	0.0	0.0		9	50.0	9	50.0	
Respiratory illness	8	80.0	2	20.0		9	90.0	1.0	10		4	40.0	6	63.5	
Diabetes	8	88.9	1	11.1		8	88.9	1	11.1		4	44.4	5	55.6	
**History of anxiety and/or depression**															
Yes	66	84.6	12	15.4	0.057*	74	94.9	4	5.1	1.000F	21	29.6	57	73.1	0.027*
No	239	91.9	21	8.1		247	95.0	13	5.0		106	40.8	154	59.2	

F, Fisher’s *p*-value.

* , *p*-value < 0.1

**TABLE 4:** Association between personal protective equipment, contact with COVID-19 cases and infection and the dependent variables.

Variables	Depression					Anxiety					Stress				
	No	%	Yes	%	*p*	No	%	Yes	%	*p*	No	%	Yes	%	*p*
**Access to PPE**															
Yes	277	90.2	30	9.8	1.000F	292	95.1	15	4.9	0.662F	120	39.1	187	60.9	0.071
No	28	90.3	3	9.7		29	93.5	2	6.5		7	22.6	24	77.4	
**Adequacy of PPE**															
Adequate	117	90.0	13	10.0	0.908	122	93.8	8	6.2	0.455	52	40.0	78	60.0	0.467
Inadequate	188	90.4	20	9.6		199	95.7	9	4.3		75	36.1	133	63.9	
**Protective measures**															
Adequate	109	92.4	9	7.6	0.398F	112	94.9	6	5.1	0.408F	48	40.7	70	59.3	0.134F
Insufficient	191	89.3	23	10.7		204	95.3	10	4.7		79	36.9	135	63.1	
None	5	83.3	1	16.7		5	83.3	1	16.7		0	0.0	6	100.0	
**Contact with a suspected case**															
Yes	218	92.0	19	8.0	0.098*	228	96.2	9	3.8	0.112	90	38.0	147	62.0	0.816
No	87	86.1	14	13.9		93	92.1	8	7.9		37	36.6	64	63.4	
**Contact with a confirmed case**															
Yes	161	89.4	19	10.6	0.600	172	95.6	8	4.4	0.599	71	39.4	109	60.6	0.449
No	144	91.1	14	8.9		149	94.3	9	5.7		56	35.4	102	64.6	
**Contact with suspected or confirmed specimen**															
Yes	66	84.6	12	15.4	0.057*	76	97.4	2	2.6	0.379F	24	30.8	54	69.2	0.157
No	239	91.9	21	8.1		245	94.2	15	5.8		103	39.6	157	60.4	
**Got infected with COVID-19**															
Yes	49	86.0	8	14.0	0.233	55	96.5	2	3.5	0.748F	12	21.1	45	78.9	0.005*
No	256	91.1	25	8.9		266	94.7	15	5.3		115	40.9	166	59.1	
**A colleague got infected with COVID-19**															
Yes	244	90.4	26	9.6	0.869	256	94.8	14	5.2	1.000F	108	40.0	162	60.0	0.066*
No	61	89.7	7	10.3		65	95.6	3	4.4		19	27.9	49	72.1	
**A colleague died from COVID-19**															
Yes	102	85.7	17	14.3	0.039*	113	95.0	6	5.0	1.000	38	31.9	81	68.1	0.114
No	203	92.7	16	7.3		208	95.0	11	5.0		89	40.6	130	59.4	
**A family member got infected with COVID-19**															
Yes	57	82.6	12	17.4	0.017*	65	94.2	4	5.8	0.758F	23	33.3	46	66.7	0.415
No	248	92.2	21	7.8		256	95.2	13	4.8		104	38.7	165	61.3	
**A family member died of COVID-19**															
Yes	16	84.2	3	15.8	0.414F	18	94.7	1	5.3	1.000F	5	26.3	14	73.7	0.297
No	289	90.6	30	9.4		303	95.0	16	5.0		122	38.2	197	61.8	
**Afraid of getting infected with COVID-19**															
Yes	237	90.5	25	9.5	0.799	252	96.2	10	3.8	0.058*	96	36.6	166	63.4	0.511
No	68	89.5	8	10.5		69	90.8	7	9.2		31	40.8	45	59.2	
**Willingness to work as a frontline staff**															
Yes	145	89.0	18	11.0	0.444	154	94.5	9	5.5	0.690	57	35.0	106	85.0	0.340
No	160	91.4	15	8.6		167	95.4	8	4.6		70	40.0	105	60.0	

PPE, Personal Protective Equipment; F, Fisher’s *p*-value. COVID-19, coronavirus disease 2019.

Bolded *p*-values are significant at < 0.1.

* , *p*-values ρ 0.1

**TABLE 5:** Binary logistic regression analysis of covariates and depression.

Variables	AOR	Coefficient	95%	CI	*p*
**Age**					
20–29 years (Reference)	1	-	-	-	-
30–39	0.581	−0.543	0.235	1.436	0.240
≥ 40	0.224	−1.498	0.057	0.876	0.032*
**Occupation**					
Doctor (Reference)	1	-	-	-	-
Nurse	3.956	1.375	1.194	13.103	0.024*
**Days worked per week during pandemic**					
1–2 (Reference)	1	-	-	-	-
3–4	0.392	−0.936	0.140	1.098	0.075
≥ 5	0.954	−0.047	0.299	3.047	0.937
**History of anxiety and/or depression**					
No (Reference)	1	-	-	-	-
Yes	1.802	0.589	0.749	4.336	0.189
**Contact with a suspected case**					
Yes (Reference)	1	-	-	-	-
No	2.313	0.839	0.895	5.976	0.083
**Contact with suspected or confirmed specimen**					
No (Reference)	1	-	-	-	-
Yes	2.504	0.918	1.033	6.070	0.042*
**A colleague died of COVID-19**					
No (Reference)	1	-	-	-	-
Yes	2.912	1.069	1.210	7.004	0.017*
**A family member got infected**					
No (Reference)	1	-	-	-	-
Yes	2.659	0.978	1.128	6.265	0.025*

AOR, Adjusted Odds Ratio. COVID-19, coronavirus disease 2019.

Hosmer and Lemeshow *χ*^2^ (*p*-value): 6.308 (0.613); Nagelkerke *R*^2^: 0.223.

* , *p*-value <0.05

The two variables associated with anxiety at bivariate analysis retained their significant power at regression analysis. Nurses, compared with doctors, were 6.82 times more likely (95% CI: 1.360–34.157) to experience anxiety. Healthcare professionals that were not afraid of contracting COVID-19 were 3.12 times (95% CI: 1.022–9.510), more likely to experience anxiety (Table 6). The independent variables explained 11.9% of the variance.

**TABLE 6:** Binary logistic regression analysis of covariates and anxiety.

Variables	AOR	Coefficient	95%	CI	*p*
**Age**					
20–29 years (Reference)	1	-	-	-	-
30–39	0.798	−0.226	0.263	2.421	0.690
≥ 40	0.388	−0.947	0.074	2.035	0.263
**Gender**					
Male (Reference)	1	-	-	-	-
Female	0.536	−0.623	0.173	1.659	0.279
**Occupation**					
Doctor (Reference)	1	-	-	-	-
Nurse	6.815	1.919	1.360	34.157	0.020*
**A colleague got infected with COVID-19**					
Yes (Reference)	1	-	-	-	-
No	0.491	−0.711	0.121	1.989	0.319
**A colleague died of COVID-19**					
Yes (Reference)	1	-	-	-	-
No	0.843	−0.171	0.275	2.581	0.765
**A family member got infected**					
No (Reference)	1	-	-	-	-
Yes	0.895	−0.111	0.222	3.615	0.876
**A family member died of COVID-19**					
Yes (Reference)	1	-	-	-	-
No	1.435	0.361	0.128	16.042	0.769
**Afraid of getting infected with COVID-19**					
Yes (Reference)	1	-	-	-	-
No	3.118	1.137	1.022	9.510	0.046*

AOR, Adjusted Odds Ratio COVID-19, coronavirus disease 2019.

Hosmer and Lemeshow *χ*^2^ (*p*-value): 8.120 (0.422); Nagelkerke *R*^2^: 0.119.

* , *p*-value <0.05

Age, occupation, a history of depression or anxiety and COVID-19 infection were identified as factors associated with stress among the HCPs. Healthcare professionals that were ≥ 40 years were less likely (95% CI: 0.147–0.981) to be stressed compared with their younger counterparts. This implies that younger HCPs (20–29 years) were 2.64 times more likely to be stressed (reversed reference). Nurses were more likely to be stressed (OR: 2.35; 95% CI: 1.388–3.974) than doctors. Those with a history of anxiety or depression were almost inevitably bound to be stressed compared to those without such history. Stress became more significant, up to 3.33 times more probable (95% CI: 1.599–6.935) if the HCPs had been infected with COVID-19 (Table 7). The independent variables explained 17.7% of the variance.

**TABLE 7:** Binary logistic regression analysis of covariates and stress.

Variables	AOR	Coefficient	95%	CI	*p*
**Age**					
20–29 years (Reference)	1	-	-	-	-
30–39	0.505	−0.684	0.254	1.001	0.050
≥ 40	0.379	−0.969	0.147	0.981	0.046*
**Occupation**					
Doctor (Reference)	1	-	-	-	-
Nurse	2.349	0.854	1.388	3.974	0.001*
**Marital status**					
Married (Reference)	1	-	-	-	-
Not-married	1.594	0.466	0.812	3.128	0.175
**Whom you live with**					
Alone (Reference)	1	-	-	-	-
With family	0.552	−0.594	0.267	1.144	0.110
**Years of employment**					
1–5 (Reference)	1	-	-	-	-
6–10	1.010	0.010	0.542	1.882	0.975
> 10	0.763	−0.271	0.340	1.714	0.512
**History of anxiety and/or depression**					
No (Reference)	1	-	-	-	-
Yes	1.925	0.655	1.057	3.507	0.032*
**Access to PPE**					
Yes (Reference)	1	-	-	-	-
No	2.089	0.737	0.826	5.282	0.119
**Got infected with COVID-19**					
No (Reference)	1	-	-	-	-
Yes	3.330	1.203	1.599	6.935	0.001*
**A colleague got infected with COVID-19**					
Yes (Reference)	1	-	-	-	-
No	1.375	0.318	0.714	2.646	0.341

AOR, Adjusted Odds Ratio; PPE, personal protective equipment. COVID-19, coronavirus disease 2019

Hosmer and Lemeshow *χ*^2^ (*p*-value): 7.759 (0.457); Nagelkerke *R*^2^: 0.177.

* , *p*-value <0.05

## Discussion

This study assessed the prevalence and factors associated with depression, anxiety and stress among clinical HCPs working in a tertiary hospital in Nigeria. The prevalence of depression, anxiety and stress was 9.8%, 5.0% and 62.4%, respectively. Comparing the findings in this study to other studies in Africa is challenging because of the paucity of studies on the mental health of healthcare workers since the COVID-19 pandemic. A recent systematic review conducted by^28^ found no African studies. Since their review, however, some studies have emerged from Africa.^13,14,15^ The prevalence of depression among HCPs in our study is significantly lower than the 21% reported in Ghana^15^ and 30.5% reported in Tunisia.^14^ In comparison to studies outside Africa, our finding is still lower than the pooled depression prevalence of 21.7% in a recent systematic review,^28^ 18.3% reported in China and 42.3% reported in Trinidad and Tobago.^11,29^

The prevalence of anxiety of 5% found in this present study is significantly lower than 22.2% – 27.8% reported in Africa.^14,15^ Also, it is lower than the pooled anxiety prevalence of 22.1% across 57 countries,^28^ as well as 11.0% reported in Saudi Arabia,^30^ 12.3% in China,^7^ 20.7% in Spain,^9^ 25.9% in Oman^31^ and 33% in the United States.^8^ The prevalence of stress was 62.4% in our study, which is similar to the 56.4% reported in Oman,^31^ and considerably higher than the 8.2% in a study conducted among Ghanaian health workers,^15^ 17.9% in Trinidad and Tobago^11^ and 18.6% in Tunisia.^14^ The plausible explanations for the variation in the prevalence of depression, anxiety and stress can be linked to the assessment tools used in these studies and the time the studies were conducted.^15^ While some studies were conducted close to the peak of the pandemic, other studies were conducted nearly a year later – such as the current study.

Several measures were adopted by the HCPs to cope with COVID-19 and associated stress. An increase in knowledge about COVID-19, contributed to a more positive attitude towards working with COVID-19 patients. Respondents also used preventive measures (such as regular hand washing and use of face masks) to cope with stress. Earlier studies are in agreement with this finding.^15,16^

Preventive measures against COVID-19 are extremely important, more so for HCPs that treat patients with COVID-19. Almost all the HCPs used social engagement such as regular contact via social media with family and friends to cope with symptoms of depression, anxiety and stress. This is similar to findings reported in earlier studies.^14,15^ Interpersonal contact is seen as a mitigating factor to alleviate anxiety and depression to improve the mood of the HCPs.^32^ About one in ten of the HCPs in this study stopped watching COVID-19-related news and two-thirds resorted to spiritual means to assist them to cope with the stress of COVID-19, findings that are in agreement with an earlier study.^15^

Healthcare professionals at least 40 years of age, compared to those between 20 years and 29 years, were found to have less symptoms associated with depression and stress. A study conducted in China reported an inverse of our findings.^2^ A plausible explanation for our findings could be that older HCPs play more of a supervisory or clinical instructor role, thereby limiting their contact with COVID-19 patients. In addition, there were concerns about the adequacy of the protective equipment available in the facility by more than half of the HCPs (see Table 2); this may contribute to depression among the younger HCPs as they are likely to have more contact with COVID-19 patients while providing care. Healthcare professionals whose colleagues and relatives died because of complications relating to COVID-19 were more likely to be depressed in our study. Some of the reasons include the affected HCPs might still find it difficult to cope with the deaths of their colleagues; unresolved grieve might lead to depression,^33^ especially if the deceased is a close family member. Another reason might be that the fear of contracting COVID-19 may increase if a colleague or family member dies of COVID-19, which might lead to concerns over the possibility of contracting COVID-19, making one vulnerable to stress, anxiety or depression.^34^

We found that nurses were significantly more depressed and experienced anxiety-related symptoms than doctors. This is in line with the conclusion of Pappa et al.^35^ in their systematic review and meta-analysis as they reported that nurses presented with significantly greater anxiety-related features than doctors. A similar observation was reported in Switzerland,^36^ Spain^9^ and China.^2^ The variation in the level of anxiety between both professionals can be attributed to two possible reasons. Firstly, nurses tend to have more contact with infected patients because they spend more time in the ward in direct contact with the patients, collecting specimens.^2,9^ Secondly, nurses may witness more injuries including ethical dilemmas and death, which can contribute to their high level of anxiety.^2,35^ Therefore, there is a need to focus on interventions for nurses to alleviate their anxiety as suggested by Zhu et al.^37^ Healthcare professionals that were afraid of getting infected with the virus had a very high probability of experiencing anxiety. Perhaps the fear of COVID-19 infection and witnessing frequent mortality may have led to the high levels of anxiety.^2^

One study affirms our finding that older HCPs were less likely to be stressed.^9^ The study stated that younger HCPs were also likely to be junior in rank, attending to patients directly, while the older HCPs work primarily in a supervisory capacity. In addition, the lack of experience with COVID-19 patients may further increase the level of stress among the younger HCPs.^9^ Our findings point to the fact that nurses were more stressed than doctors. In addition to spending more time caring for COVID-19 patients, nurses also had to provide social and emotional support in the absence of family members, which might have increased the likelihood of stress among the nurses.^8^ The HCPs with a history of anxiety and/or depression experienced significantly more stress. A history of stress is generally seen as a predisposing factor for anxiety or depression, which was proven in this study. Healthcare professionals that returned to work after recovering from COVID-19 were more stressed than those who never tested positive for COVID-19. The stress could be induced by the circumstances related to the recovery from COVID-19 such as isolation from family and colleagues.

## Strength and limitations

Many of the previous studies selected participants with non-probability sampling such as convenience and consecutive sampling. A major strength of our study is the use of probability sampling to limit sampling bias. Also, this study is one of the few available studies on COVID-19-related mental health among HCPs in Nigeria. This study is not without limitations. Firstly, only one health facility was surveyed, which may not be a representation of the mental health status of HCP in Lagos State and Nigeria at large; more studies across the country are warranted. Secondly, causal effect cannot be established as this is a cross-sectional study; therefore, the findings should be interpreted with caution. Thirdly, self-administered closed-ended questions may have limited the extent of experience of the HCPs; we recommend a qualitative study to assess the lived-in experience of HCPs during the peak of COVID-19. Finally, the low Cronbach alpha of the PSS tool suggests that the scale is less reliable in this setting; therefore, the findings should be interpreted with some caution. Further, there is a need for the PSS tool to be validated in the Nigerian setting.

## Conclusions

Nurses experienced the most stress and were therefore identified as a vulnerable group, more at risk to develop anxiety and depression. Psychosocial interventions and stress management techniques are recommended to minimise the risks.
